# Reducing cognitive load in choral rehearsal: evidence-informed conducting pedagogies based on music perception theory

**DOI:** 10.3389/fpsyg.2026.1825435

**Published:** 2026-06-18

**Authors:** Yuqiong Zhang, Yiming Lyu, Danyang Hou

**Affiliations:** College of Music, Sookmyung Women's University, Seoul, Republic of Korea

**Keywords:** choral rehearsal, cognitive load, conducting gestures, music perception, rehearsal effectiveness

## Abstract

The purpose of this investigation was to determine whether varying degrees of conducting gesture complexity would influence choral musicians' perceived cognitive load and their evaluations of rehearsal effectiveness. Two choral directors were recorded leading 1-min segments from Eric Whitacre's *Lux Aurumque* using simplified, standard, and elaborated conducting gestures. These six recordings were then paired with an identical high-quality choral audio track or a separate sight-reading audio excerpt. University choral ensemble participants (*N* = 120; novice, *n* = 60; experienced, *n* = 60) viewed all six stimulus recordings and assessed both their perceived cognitive load and the effectiveness of the rehearsal process. Participants also composed one brief written observation about either the conductor or the rehearsal after each video. Findings revealed that gesture complexity had a statistically significant impact on ratings of both cognitive load and rehearsal effectiveness. A significant interaction emerged between gesture complexity (simplified, standard, and elaborated) and rehearsal context (sight-reading or polishing) with respect to cognitive load assessments. Participants indicated that the elaborated gesture condition during sight-reading imposed the highest cognitive demands. The standard gesture approach received the most favorable effectiveness ratings regardless of rehearsal context. Written observations predominantly addressed conductor behaviors rather than rehearsal outcomes, with the elaborated-gesture recordings generating the highest proportion of negative responses.

## Conducting gestures and music perception

Cognitive load has attracted growing attention among scholars investigating the intersection of music pedagogy and human information processing. Sweller's (1988) cognitive load theory (CLT) posits that instructional design should account for the limited capacity of working memory, and researchers in educational psychology have consistently demonstrated that poorly calibrated instructional demands lead to diminished learning outcomes (Paas et al., 2003; Sweller et al., 1998). Within choral settings, the conductor serves simultaneously as a visual information source, an expressive model, and a temporal organizer, creating a uniquely complex perceptual environment for singers (Durrant, 2003; Garnett, 2009). Additional investigations have suggested that the cognitive resources demanded by following a conductor may vary substantially depending on the visual complexity of the gestural information presented (Byo and Austin, 1994; Wollner, 2008). More recently, research on multimodal integration in music performance has revealed that auditory processing can be significantly modulated by concurrent visual stimuli (Platz and Kopiez, 2012; Tsay, 2013).

Although the importance of gesture in conducting is widely acknowledged, relatively few empirical studies have examined how the informational density of conducting movements affects performers' cognitive processing. Much of the extant literature on conducting effectiveness has concentrated on whether conductors appear expressive or whether audiences prefer certain nonverbal behaviors (Napoles, 2013; Price and Winter, 1991; Silvey, 2011; Whitaker, 2011). Separate lines of inquiry have confirmed that evaluations of conducting competence are shaped more strongly by perceived expressivity than by any other single attribute (Price and Chang, 2001, 2005; Silkebakken, 1988). Furthermore, the visual dimension of conducting has been shown to alter listeners' judgments of ensemble performance quality even when the audio stimulus remains constant (Morrison and Selvey, 2014; Morrison et al., 2009; Price and Mann, 2011). Taken together, these findings suggest that what a conductor does visually exerts a powerful influence on how both the conductor and the ensemble are evaluated.

The gestures that are conducted are not a linear progression of level of difficulty but are a multi-functional system that involves: temporal coordination, expressive communication, and instruction/signaling. These functions do not act in isolation, but rather all need to be executed simultaneously by performers with the possibility of cognitive interference when multiple streams of gestural information compete to be paid attention. The amount of cognitive load that is required for communicating a gesture is not only problem solving, but also the function of the gesture, the congruence with the musical content and its rehearsal setting.

Despite these insights, investigators have not yet established a consistent link between the complexity of conducting gestures and singers' self-reported cognitive demands during rehearsal. Price and Chang (2001, 2005) observed that music majors' commentary underscored the salience of expressivity in their overall conductor appraisals, yet neither study addressed the cognitive costs that elaborate gestural information might impose on the performers themselves. Comparable findings were reported by Price (2006), whose data indicated that undergraduate music students' ratings of conductor video-only segments did not predict their concurrent evaluations of ensemble sound quality. The numerous aural and visual elements that shape perceptions of conducting—including body type, repertoire, gesture vocabulary, and gender—render it difficult to isolate the cognitive consequences of any single dimension.

## Gestural complexity in conducting pedagogy

One dimension of conducting pedagogy that may bear directly on performers' cognitive engagement, yet has received scant empirical scrutiny, concerns the complexity of the gestures a director employs during rehearsal. Several prominent conducting method texts offer recommendations about the appropriate level of gestural detail for different instructional contexts, ranging from stripped-down beat patterns intended to prioritize clarity (Green and Gibson, 2004; Labuta, 1982) to richly layered movements designed to model phrasing, dynamics, and articulation simultaneously (Garofalo and Battisti, 2005; Jordan, 2009). A comprehensive survey of conducting textbooks reveals three broad categories of gestural complexity: (a) simplified gestures, which emphasize metrical clarity through economical motion (Green and Gibson, 2004; Hunsberger and Ernst, 1992; Rudolf, 1994); (b) standard gestures, incorporating moderate expressive shaping alongside clear beat patterns (Garofalo and Battisti, 2005; Jordan, 2009; Phillips, 1997); and (c) elaborated gestures, featuring extensive left-hand independence, facial modeling, and continuous dynamic contouring (Eichenberger, 1994; Neuen, 2002; Stanton, 1971). Although each approach has its advocates, none of the referenced sources provided empirical support for their recommendations.

Advocates of simplified conducting typically emphasize the need for rhythmic precision and ensemble synchronization, particularly during initial reading sessions when singers are still decoding notation (Green and Gibson, 2004). Authors who favor the standard approach generally argue that a balance between clarity and expressiveness facilitates both accuracy and musical engagement without overwhelming performers (Garofalo and Battisti, 2005). Meanwhile, proponents of elaborated conducting contend that the richness of the gestural vocabulary directly models the desired musical outcome, thereby reducing the need for verbal instruction (Eichenberger, 1994; Neuen, 2002). Neuen (2002) specifically cautioned that overly restrained arm movements appear “expressively very limited” (p. 216), while Eichenberger (1994) advocated for a holistic gestural approach that integrates breath, dynamics, and articulation into a single visual stream. Notwithstanding these strong pedagogical opinions, none of the authors cited experimental evidence to substantiate claims about how performers actually process the visual information contained in conducting gestures.

## Cognitive load theory as a theoretical lens: purpose and research questions

Cognitive load theory provides a principled framework for examining this question. According to CLT, the total demand placed on working memory during any learning task comprises three components: intrinsic load, which stems from the inherent complexity of the material; extraneous load, which arises from suboptimal instructional design; and germane load, which reflects the mental effort devoted to constructing durable schemas (Sweller et al., 1998). In a choral rehearsal, intrinsic load is determined largely by the difficulty of the music being rehearsed, whereas extraneous load may be influenced by the clarity or complexity of the conductor's gestural communication. If elaborated gestures provide information that singers cannot readily integrate with the auditory and notational demands of the music, the resulting extraneous load may impair rather than enhance rehearsal effectiveness.

To date, no published investigation has systematically varied conducting gesture complexity while holding musical content constant and measuring both perceived cognitive load and rehearsal effectiveness. Nor has prior research examined whether the rehearsal context—specifically, whether singers are sight-reading unfamiliar material or polishing a previously learned passage—moderates the relationship between gestural complexity and cognitive demand. Understanding these dynamics could inform conducting pedagogy by helping directors calibrate their gestural vocabulary to the informational needs of their ensemble at different stages of the rehearsal process. In addition to assessing whether gesture complexity influences choral musicians' perceptions of cognitive load and rehearsal effectiveness, we sought to compare responses between novice and experienced choral singers. The present investigation was designed to address the following research questions:

(1) Would university choral musicians report different levels of perceived cognitive load after viewing conductors who demonstrated three distinct gesture complexity levels (i.e., simplified, standard, and elaborated) across two rehearsal contexts (sight-reading, polishing)?
(2) Would there be differences between novice and experienced choral singers' ratings of cognitive load and rehearsal effectiveness based upon gesture complexity and the type of rehearsal context observed?

## Methods

### Conductor and musical stimulus preparation

Our methodology drew upon recent investigations of conductor–ensemble interaction in which visual aspects of conducting behavior were systematically manipulated and the resulting videos were synchronized with fixed audio recordings (Morrison et al., 2009; Morrison and Selvey, 2014; Silvey, 2011, 2013). We engaged two graduate conducting students specializing in choral music to serve as the conductor models for the stimulus materials (both female, ages 26 and 31). Both conductors were enrolled at the first author's institution, had directed community or church choirs for at least 3 years prior to beginning their graduate studies, and had participated in advanced choral conducting workshops.

During an initial orientation session, each conductor received a professional recording of mm. 22–40 of Eric Whitacre's *Lux Aurumque* for mixed chorus. We selected this excerpt because it features sustained homophonic textures, gradual dynamic shifts, demands careful attention to vowel unification, and lasts approximately 1 min in duration. Selection of this musical passage was consistent with prior stimulus-based conducting research in that the excerpt was slow, lyrical, and required careful attention to phrasing and blend (Morrison et al., 2009; Price, 2006; Silvey, 2013).

Beyond the recording, we instructed each conductor to practice leading the excerpt at three levels of gesture complexity. In the simplified condition, conductors maintained a clear, metronomic beat pattern with minimal left-hand independence and neutral facial expression. In the standard condition, they incorporated moderate dynamic shaping, occasional left-hand cueing, and subtle facial expression that corresponded to the musical character. In the elaborated condition, conductors employed continuous left-hand modeling, extensive dynamic contouring, pronounced facial expression mirroring the emotional content, and visible breath cues. A researcher-developed rubric operationally distinguishing the three complexity levels, grounded in descriptions found in prominent conducting textbooks, was provided to each conductor (Eichenberger, 1994; Green and Gibson, 2004; Neuen, 2002).

The conductors were given 1 week to internalize the excerpt and rehearse conducting at each complexity level. At a follow-up session, each conductor successfully demonstrated all three complexity conditions in synchronization with the choral audio track. Both conductors were then scheduled for recording sessions the following week.

### Recording and stimulus assembly

Frontal-view recordings of each conductor were captured in two separate rehearsal spaces on the university campus where the participants were enrolled. Both conductors wore similar professional concert attire while simulating performances of the assigned excerpt. The camera field was framed to show each conductor from approximately the waist upward. Three separate video recordings—each featuring one of the three gesture complexity levels—were made of each conductor. To confirm that the intended complexity distinctions were maintained and perceptible, we showed the finished videos to two choral conducting faculty members who concurred that (a) the differences among gesture complexity conditions were readily distinguishable and (b) the musical phrasing implied by each conductor remained consistent across conditions.

After importing the recordings into Adobe Premiere Pro 2023, we removed the original audio from each of the six video files. This method enabled control of the auditory input, but didn't capture the interactive aspects of gesture-speech interaction in real rehearsal contexts. This is a trade-off between experimental control and ecological validity, which actually sacrifices some of the latter to isolate some of the visual variables. We then created two sets of synchronized stimuli: one set paired each conductor's three videos with a high-quality professional choral recording of the same excerpt (the polishing condition), while a parallel set paired the identical videos with a separate audio recording of an advanced university choir sight-reading the passage for the first time (the sight-reading condition). The sight-reading audio was selected because it contained minor pitch and rhythmic imprecisions characteristic of an initial reading, providing ecological validity for the sight-reading rehearsal context. Using Latin-square counterbalancing to mitigate order effects, we arranged the six videos into six presentation sequences such that no conductor or complexity level appeared consecutively. Each sequence was uploaded as a private playlist on a secure institutional streaming platform for subsequent evaluation.

### Participants and procedure

Participants (*N* = 120) were choral ensemble members enrolled at two midsized university music programs. The sample was divided evenly between novice choristers (*n* = 60), defined as those with fewer than four semesters of collegiate choral experience, and experienced choristers (*n* = 60), who had completed four or more semesters. The sample was distributed relatively evenly by gender (male, *n* = 58; female, *n* = 62). Data collection occurred during a combined rehearsal day at one of the two institutions. Informed-consent documents were distributed and reviewed before evaluation forms were administered.

Participants were read standardized instructions before viewing the six stimulus videos. There was no active singing and participants had to rate prerecorded stimuli. It is, therefore, a study based on perceptions, not performer responses. After each video, participants first rated their perceived cognitive load on a 10-point Likert-type scale anchored by *very low mental effort* (1) and *very high mental effort* (10). Judged the effectiveness of the rehearsal, defined here as a subjective evaluation of how clearly the conductor's gestures appeared to facilitate coordination and musical direction within the excerpt. Additionally, participants wrote one brief comment about either the conductor's gestures or the rehearsal outcome after each of the six videos. By incorporating this written forced-choice task, we aimed to reveal participants' primary attentional focus—whether visual or aural—while viewing the stimuli. Completed evaluation forms were collected at the conclusion of the approximately 15-min evaluation session.

## Results

### Perceived rehearsal effectiveness

To maximize interpretive clarity, we treated each instance of rehearsal context and gesture complexity as a within-subjects repeated measure (Tabachnick and Fidell, 1989).

Rehearsal effectiveness ratings for each of the six stimulus videos (conductor A: simplified, standard, and elaborated gestures; conductor B: simplified, standard, and elaborated gestures) were analyzed using a three-way mixed ANOVA with one between-subjects factor (musician experience) and two within-subjects factors (rehearsal context and gesture complexity). Table 1 presents the mean responses and standard deviations for rehearsal effectiveness by musician experience (novice or experienced), rehearsal context (sight-reading or polishing audio), and gesture complexity (simplified, standard, and elaborated). Prior to computing the three-way mixed ANOVA, a separate repeated-measures ANOVA confirmed no presentation order effect, *F*_(5, 595)_ = 0.73, *p* = 0.60.

**Table 1:** Descriptive statistics for perceived rehearsal effectiveness by gesture complexity, musician experience, and rehearsal context.

Gesture complexity	Rehearsal context			
	Sight-reading		Polishing	
	*M*	*SD*	*M*	*SD*
Novice choristers (*n* = 60)				
Simplified	7.65	1.52	7.48	1.45
Standard	8.12	1.38	7.95	1.57
Elaborated	6.78	1.81	7.03	1.69
Experienced choristers (*n* = 60)				
Simplified	7.82	1.37	7.55	1.62
Standard	7.93	1.48	8.08	1.41
Elaborated	6.95	1.74	6.87	1.88
Overall (*N* = 120)				
Simplified	7.73	1.44	7.52	1.53
Standard	8.03	1.43	8.02	1.49
Elaborated	6.87	1.77	6.95	1.78

Because the sphericity assumption was violated (χ^2^ = 10.84, *p* = 0.004), Greenhouse–Geisser corrected degrees of freedom were applied to the gesture complexity factor. No main effect was detected for the between-subjects factor, musician experience, *F*_(1, 118)_ = 0.18, *p* = 0.67, and partial η^2^ = 0.002, or for the within-subjects factor, rehearsal context, *F*_(1, 118)_ = 0.95, *p* = 0.33, and partial η^2^ = 0.008. A main effect emerged for the within-subjects factor of gesture complexity, *F*_(1.81, 213.58)_ = 24.67, *p* < 0.001, partial η^2^ = 0.17. Bonferroni-corrected *post hoc* comparisons revealed significantly higher (*p* < 0.05) rehearsal effectiveness ratings for both the standard (*M* = 8.03, *SD* = 1.46) and simplified (*M* = 7.63, *SD* = 1.49) gesture conditions compared with the elaborated condition (*M* = 6.91, *SD* = 1.78). No significant interactions were detected among any combination of the variables examined.

### Perceived cognitive load

Cognitive load ratings for each of the six stimulus videos were compared using a parallel three-way mixed ANOVA with one between-subjects factor (musician experience) and two within-subjects factors (rehearsal context and gesture complexity). Table 2 displays mean responses and standard deviations for cognitive load by musician experience (novice or experienced), rehearsal context (sight-reading or polishing), and gesture complexity (simplified, standard, and elaborated). A separate repeated-measures ANOVA confirmed no order effect, *F*_(5, 595)_ = 1.18, *p* = 0.32.

**Table 2:** Descriptive statistics for perceived cognitive load by gesture complexity, musician experience, and rehearsal context.

Gesture complexity	Rehearsal context			
	Sight-reading		Polishing	
	*M*	*SD*	*M*	*SD*
Novice choristers (*n* = 60)				
Simplified	4.02	1.71	4.18	1.52
Standard	5.48	1.72	5.32	1.81
Elaborated	7.38	1.53	6.52	1.78
Experienced choristers (*n* = 60)				
Simplified	3.62	1.76	3.88	1.59
Standard	5.15	1.63	5.03	1.69
Elaborated	6.92	1.62	6.25	1.85
Overall (*N* = 120)				
Simplified	3.82	1.74	4.03	1.56
Standard	5.32	1.67	5.18	1.75
Elaborated	7.15	1.58	6.38	1.82

**Figure 1:**
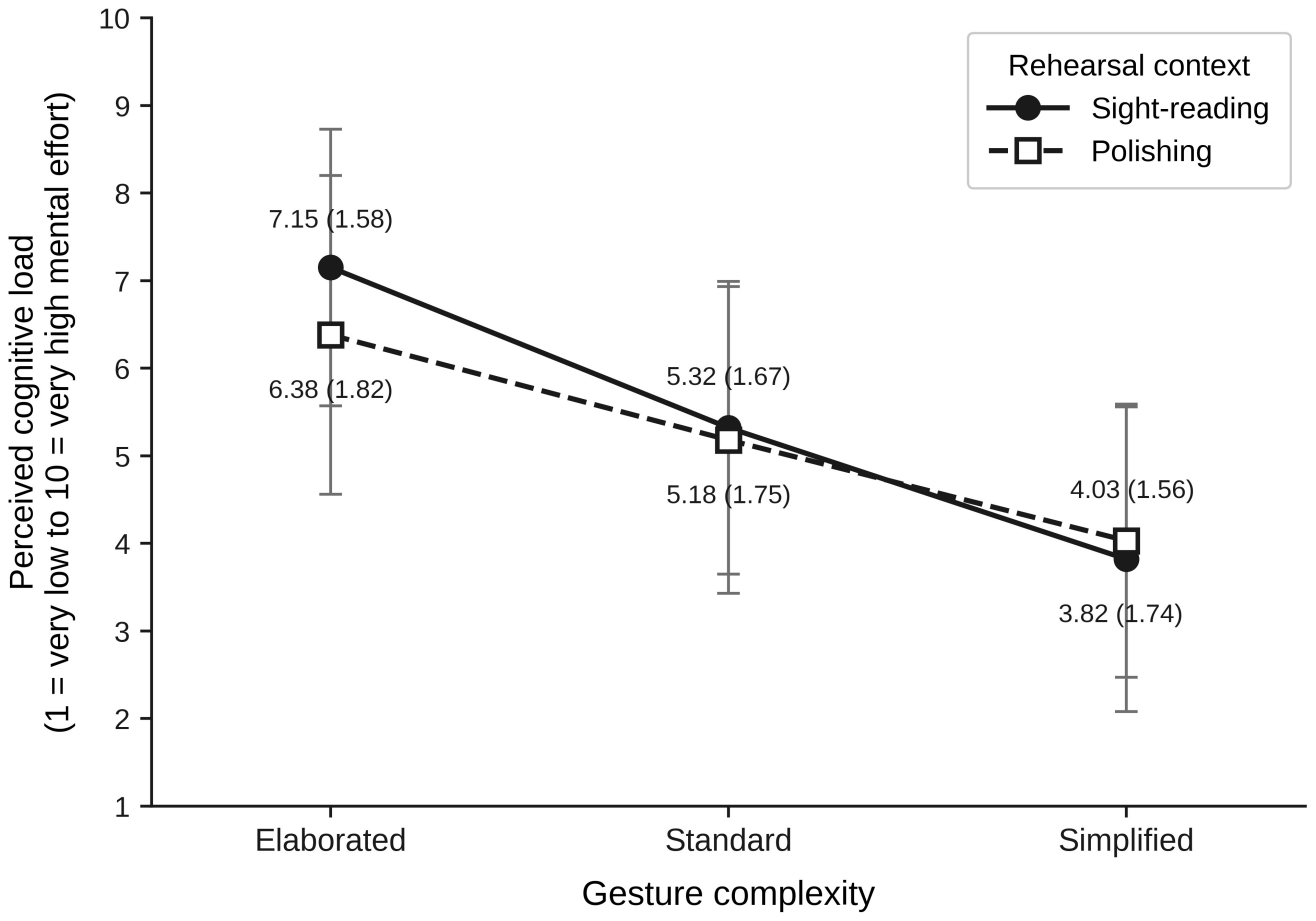
Means (standard deviations) for cognitive load ratings by rehearsal context and gesture complexity. Ratings were anchored by very low mental effort (1) and very high mental effort (10).

Greenhouse–Geisser corrected degrees of freedom were again used for the gesture complexity factor due to a sphericity violation (χ^2^ = 8.92, *p* = 0.012). No main effect was found for musician experience, *F*_(1, 118)_ = 0.31, *p* = 0.58, η^2^ = 0.003, or for rehearsal context, *F*_(1, 118)_ = 2.78, *p* = 0.10, and partial η^2^ = 0.02. A main effect was found for gesture complexity, *F*_(1.84, 217.12)_ = 52.36, *p* < 0.001, and partial η^2^ = 0.31. Bonferroni *post hoc* testing indicated significantly higher (*p* < 0.05) cognitive load ratings for the elaborated condition (*M* = 6.77, *SD* = 1.70) than for either the standard (*M* = 5.25, *SD* = 1.71) or simplified (*M* = 3.93, *SD* = 1.65) conditions. No significant interactions were detected for Rehearsal Context × Musician Experience, *F*_(1, 118)_ = 0.85, *p* = 0.36, and partial η^2^ = 0.007; Gesture Complexity × Musician Experience, *F*_(2, 217.12)_ = 0.34, *p* = 0.71, partial η^2^ = 0.003; or Rehearsal Context × Gesture Complexity × Musician Experience, *F*_(2, 231.48)_ = 0.92, *p* = 0.40, partial η^2^ = 0.008. A statistically significant interaction was detected for Rehearsal Context × Gesture Complexity, *F*_(2, 236)_ = 6.53, *p* = 0.002, partial η^2^ = 0.05. As depicted in Figure 1, participants reported the highest cognitive load when viewing elaborated gestures during the sight-reading context. In the polishing condition, the difference in cognitive load between elaborated and standard gestures was slightly narrower than in the sight-reading condition. The simplified gesture condition yielded the lowest cognitive load ratings in both rehearsal contexts, with the polishing context producing slightly higher reports than sight-reading—a pattern opposite to that observed in the elaborated condition.

### Participants' written comments

We employed coding protocols comparable to those used by Price and Mann (2011) and Silvey (2011) in classifying participants' written remarks. Comments (*N* = 787) were sorted by category (rehearsal process or conductor behavior), gesture complexity condition (simplified, standard, or elaborated), and valence (positive, negative, or neutral). This procedure yielded seven rehearsal-process categories and eight conductor-behavior categories. All response categories are presented in Table 3. To establish intercoder reliability, the first author analyzed all comments; the second author independently coded 25% of the total corpus. Interrater agreement was high for both category assignment (*r* = 0.91) and comment valence (*r* = 0.93), so the first author's codings were retained for all subsequent analyses.

**Table 3:** Categorization of comments for simplified, standard, and elaborated gesture conditions.

Category	Simplified (*n* = 255)				Standard (*n* = 262)				Elaborated (*n* = 270)			
	Pos	Neg	Neu	Σ%	Pos	Neg	Neu	Σ%	Pos	Neg	Neu	Σ%
Rehearsal process												
Accuracy	35	8	2	17.65	28	14	3	17.18	11	19	0	11.11
Tone quality	2	0	0	0.78	1	1	0	0.76	0	2	0	0.74
Phrasing	8	2	1	4.31	12	3	2	6.49	4	5	1	3.70
Rhythm	1	0	0	0.39	0	1	0	0.38	1	2	0	1.11
Engagement	3	0	0	1.18	5	0	1	2.29	1	0	0	0.37
Concentration	1	2	0	1.18	2	1	1	1.53	0	3	0	1.11
Ensemble cohesion	0	1	0	0.39	1	0	0	0.38	0	1	0	0.37
Conductor behavior												
Clarity	28	5	3	14.12	22	10	2	12.98	9	25	1	12.96
Gesture complexity	6	3	1	3.92	8	5	2	5.73	12	58	3	27.04
Musicality	14	7	2	9.02	18	6	4	10.69	7	13	3	8.52
Facial expression	10	4	3	6.67	19	8	2	11.07	11	9	1	7.78
Eye contact	2	0	0	0.78	4	1	0	1.91	1	1	0	0.74
Beat pattern	11	18	7	14.12	25	12	4	15.65	15	21	8	16.30
Cueing	3	1	0	1.57	5	3	0	3.05	2	4	0	2.22
Physical tension	0	1	0	0.39	1	0	0	0.38	0	3	0	1.11
Not specific	5	3	1	3.53	8	1	3	4.58	6	4	2	4.44
No comment	0	0	8	3.14	0	0	15	5.73	0	0	12	4.44
Σ	129	55	28		159	66	37		80	170	31	
Σ%	60.85	25.94	13.21	100	60.69	25.19	14.12	100	28.47	60.50	11.03	100

Pos, positive; Neg, negative; Neu, neutral.

Although participants heard identical audio tracks, only 18.50% of comments in the elaborated condition addressed rehearsal process, with 81.50% directed at conductor behaviors. The proportion of rehearsal-to-conductor comments was more evenly distributed in the standard (29.01% vs. 70.99%) and simplified (25.88% vs. 74.12%) conditions.

Written observations were predominantly negative following the elaborated-gesture videos (60.50%). The corresponding percentages of negative responses for the simplified and standard conditions were substantially lower at 25.94% and 25.19%, respectively. Notably, 27.04% of all comments prompted by the elaborated-gesture recordings specifically referenced the conductors' excessive gestural complexity. By contrast, comments that explicitly mentioned gesture complexity accounted for only 5.73% and 3.92% of the total remarks directed at the standard and simplified conditions, respectively.

Participants' remarks following the elaborated-gesture videos centered on their strongly negative reactions to the density of visual information presented. When the over-yield of visual information happens, there is no gain in communicating clearly, but rather it seems like there will be far less efficiency. Representative comments included “way too much going on with the hands,” “the gestures were distracting and made it hard to focus on the music,” and “the conductor seemed to be working incredibly hard but I couldn't tell what she wanted from us.” In contrast to the standard and simplified conditions, comments regarding beat pattern clarity, musicality, and facial expression in the elaborated condition were more frequently negative than positive.

## Discussion

Previous research has established that identical ensemble performances receive higher expressivity ratings when accompanied by visually expressive conducting (Morrison et al., 2009; Morrison and Selvey, 2014; Price et al., 2011). A substantial body of evidence also confirms that a diverse range of evaluators—from college musicians to non-specialists—show preference for conductors whose gestures appear dynamic and communicative (see review by Sasanfar, 2012). The present investigation extended this line of inquiry in two novel directions: (a) by systematically varying the complexity of conducting gestures while controlling for musical content and (b) by examining whether the rehearsal context in which those gestures were observed—sight-reading vs. polishing—moderated their impact on perceived cognitive load and rehearsal effectiveness.

A central finding of this study was that participants evaluated the perceived effectiveness of identical rehearsal excerpts differently depending on the complexity of the conducting gestures they observed. Both the standard and simplified gesture conditions received significantly higher effectiveness ratings than the elaborated condition. This result aligns with and extends previous work demonstrating that nonverbal conducting behaviors such as gaze, facial expression, and gestural economy influence musicians' perceptions of performance quality (Morrison et al., 2009; Napoles, 2013; Silvey, 2013; Whitaker, 2011). At a broader level, the finding is consonant with music perception research demonstrating that visual stimuli exert substantial influence on auditory judgments (Platz and Kopiez, 2012; Tsay, 2013). For ensemble directors, perhaps the most salient implication is that the complexity of one's conducting gestures may shape how singers evaluate the quality and progress of a rehearsal—an idea consistent with the broader literature on conductor—ensemble interaction (Morrison et al., 2009; Morrison and Selvey, 2014; Napoles, 2013; Price et al., 2011).

Parallel to the rehearsal effectiveness results, participants assigned the highest cognitive load scores to the elaborated gesture condition. Because the audio tracks were identical and both conductors maintained appropriate musical phrasing regardless of complexity level, these data provide evidence that elaborated gestures imposed the greatest perceived cognitive demand on observers. This effect is probably the sum of several individual gestural effects, though, such as facial expression, manual cues, and dynamic shaping, as each factor may put a different kind of cognitive burden in the test takers. The significant interaction between gesture complexity and rehearsal context is particularly informative. Participants reported the most pronounced cognitive burden when elaborated gestures were paired with the sight-reading context, suggesting that the combination of unfamiliar musical material and dense visual information creates an especially taxing perceptual environment. In the polishing condition, the gap between elaborated and standard gestures narrowed somewhat, perhaps because familiarity with the music freed cognitive resources for processing additional visual information. These patterns are broadly consistent with cognitive load theory's prediction that extraneous load is most detrimental when intrinsic load is already high (Sweller et al., 1998).

It may be that the standard gesture approach receives the highest effectiveness ratings precisely because it optimizes the balance between informational richness and cognitive accessibility. When conductors employ standard complexity, they provide enough expressive guidance to support musical shaping without overwhelming performers with visual information that exceeds their processing capacity. We speculate that the character and familiarity of the music itself may further modulate preferences for specific complexity levels. Although certain conducting pedagogues (e.g., Eichenberger, 1994) have promoted elaborated gestures as the ideal approach regardless of context, our data suggest that university singers do not always perceive such gestural density as conducive to effective rehearsal.

Although no inherent quality of a particular gesture complexity level makes it universally superior, the consistent pattern of our results indicates that the degree of gestural elaboration influences both how performers experience the cognitive demands of a rehearsal and how they evaluate its productivity. Directors might profitably view their gestural vocabulary as an adjustable instructional variable that should be calibrated to the cognitive demands of the rehearsal task. Future research examining how simplified, standard, and elaborated gestures affect specific dimensions of choral performance—such as intonation accuracy, vowel uniformity, dynamic range, or blend—would contribute an important empirical layer to the current literature on conductor effectiveness.

One reason the elaborated gesture condition may have generated the highest cognitive load ratings is that the density of gestural information competed with singers' efforts to process the auditory and notational demands of the music. Cognitive load theory predicts that when the volume of extraneous visual information exceeds the threshold that working memory can accommodate, learning and performance suffer (Paas et al., 2003). Given that the sight-reading condition presumably demanded greater notational processing than polishing, the elevated cognitive load in the elaborated/sight-reading combination is consistent with this theoretical prediction. Stimulus materials incorporating varied camera angles or ensemble-perspective viewpoints would be a valuable direction for subsequent investigation, as they would more faithfully represent how singers actually experience the conductor's gestures during rehearsal.

Another plausible explanation for participants' negative response to the elaborated condition concerns their prior experience. If singers have been accustomed to working with conductors who use standard or simplified gestures, they may lack the perceptual schemas necessary to efficiently decode dense gestural information. This interpretation suggests that prolonged exposure to elaborated conducting might eventually reduce its cognitive cost, an hypothesis that merits longitudinal investigation. A practical implication for conducting pedagogues is that introducing complex gestural vocabularies gradually—rather than deploying them uniformly from the outset—may allow singers to develop the perceptual skills needed to benefit from such information without being overwhelmed.

We detected no statistically significant differences in either cognitive load or rehearsal effectiveness ratings attributable to musician experience (novice vs. experienced). It is important to note that although this finding is preliminary, previous studies have shown that experience can interact with the contextual and perceptual factors in affecting cognitive load. In other words, university choral singers with varying levels of ensemble participation held comparable perceptions about both the cognitive demands imposed by different gesture complexities and the effectiveness of the rehearsals they observed. That novice and experienced choristers responded similarly should be considered an encouraging finding, perhaps indicating that sensitivity to conducting gestures develops relatively early in a singer's ensemble career. If generalized within the context of undergraduate conducting and rehearsal technique courses, this result could support the inclusion of cognitive load principles in conducting curricula regardless of the experience level of the student conductors being trained. This finding might also bolster the case for integrating perceptual training—focused on gestural clarity and economy—into conducting pedagogy at all levels.

We acknowledge, however, that using the same musical excerpt for all stimulus conditions, albeit with two different conductors and two distinct rehearsal contexts, may be viewed as a constraint on generalizability. Although participants' rehearsal effectiveness scores were more homogeneous (i.e., within approximately one point) than their cognitive load scores, this consistency is anticipated given that participants heard identical professional-quality recordings. Additionally, effect sizes for the gesture complexity variable were moderate (Cohen, 1988) for both dependent measures (i.e., rehearsal effectiveness, partial η^2^ = 0.17; cognitive load, partial η^2^ = 0.31). Findings from this study should be interpreted with appropriate caution, and broad generalizations should not be drawn. Future investigators might consider varying the audio stimulus recordings to include repertoire of different styles, tempi, and difficulty levels.

It seems reasonable to suggest that additional guidance—perhaps integrated into early field experiences and introductory conducting courses—regarding the cognitive implications of gestural complexity could help preservice music educators develop more intentional rehearsal strategies. Designing efficient methods to help students recognize and practice calibrating their gestural vocabulary to the informational demands of the rehearsal task should be a priority for conducting instructors seeking to enhance their students' pedagogical effectiveness. Exercises that require students to conduct the same passage at each complexity level while receiving peer feedback on clarity and perceived cognitive demand might prove especially beneficial (Johnston, 1993). Similarly, choral directors might consider modulating their gestural complexity in response to the rehearsal phase: employing simplified or standard gestures during initial readings when cognitive demands are already high, and reserving elaborated gestures for polishing phases when singers have already internalized the basic musical framework.

As research on conducting continues to expand, disseminating empirical findings to practitioners remains essential. Authors of conducting method texts and those responsible for training the next generation of directors should integrate current evidence on gesture perception and cognitive load into their pedagogical recommendations. A research-informed approach to conducting pedagogy—one that accounts for the cognitive capacities of performers—holds promise for developing more effective young conductors, which in turn may enhance the musical development of the ensembles they lead.

There are some drawbacks to consider. First, the results of observer-based evaluations might not accurately capture the aspect of singers' real singing practices where the cognitive processes are embodied. Second, when the sounds are separated from the objects, then the gestures are less ecological as they occur directly affect the sound production in real rehearsals. Third, the complexity of the gesture was treated as a composite element, while individual parts of the gesture might require different types of cognition.

## Data Availability

The original contributions presented in the study are included in the article/supplementary material, further inquiries can be directed to the corresponding author.
